# Risk entanglement and the social relationality of risk

**DOI:** 10.1057/s41599-023-01668-0

**Published:** 2023-04-15

**Authors:** Christian von Scheve, Markus Lange

**Affiliations:** Freie Universität Berlin, Berlin, Germany

**Keywords:** Sociology, Social policy, Economics, Politics and international relations

## Abstract

Relational accounts of risk explain variation in risk perception through situated cognitions defining risk as a relationship between “risk objects” and “objects at risk”. We extend this approach to include not only the relational constitution of cognitive risk objects, but also of the different actors assessing risk. Risk in this perspective is relational because it establishes a link between two different cognitive objects and between two (or more) actors. We argue that this is the case when at least two actors refer to a common risk object while retaining distinct objects at risk. We call this a constellation of *risk entanglement* across actors. We illustrate our theoretical arguments using data from 68 qualitative interviews and ethnographic fieldwork in the German finance-state nexus. Our analyses indicate how risk entanglement affects and transforms the fundamental logics according to which both of these fields operate.

## Introduction

Risk pervades social life in contemporary societies. If seen as a specific quality of a situation that involves a decision with uncertain, potentially unfavorable or harmful outcomes, risk is primarily a phenomenon that is experienced by an individual decision maker or ascribed to a situation by an observer. Objective approaches to risk maintain that risk can be quantified given the probability and magnitude of a loss are known, as in many lotteries. However, in many cases, neither the extent of harm nor the likelihood of its occurrence are known and, depending on circumstances such as available information, past experiences, cognitive ability, or coping potential, the degree of perceived uncertainty and severity of outcomes will vary considerably across people.

This has given rise to understandings of risk that emphasize (inter)subjective interpretations of loss and their probabilities and consider these interpretations in the broader social and cultural context of the person and the situation. In this regard, a particularly prominent perspective has been developed by Douglas and Wildavsky (1982) who argue that risk itself is substantially defined by the structural and cultural conditions of a society and how they change and develop over time. This includes the view that subjective *perceptions* of risk and uncertainty do not simply differ across individuals, but do so systematically and in conjunction with the culture, worldviews, and practices that are germane to communities and societies. Beck’s *Risk Society* can be read along these lines when defining risk as “a systematic way of dealing with hazards and insecurities induced and introduced by modernization itself” (Beck, 1992, p. 21). COVID-19 and climate change and the different ways of interpreting and addressing these hazards across countries are prime examples (e.g., Dimitrijevska-Markoski and Nukpezah, 2023; Cambardella et al., 2020).

A more recent theoretical development has aimed at reconciling this cultural and macro-social perspective with the more individualized views dominant in economics and psychology (see Hansson, 2010, for a comprehensive discussion). This “relational theory of risk” argues that risk emanates from a relation between “risk objects” and “objects at risk” (Boholm and Corvellec, 2011). Briefly, risk objects are things or events in the world individuals perceive to be hazardous (e.g., criminal offenses, natural disasters, smoking) whereas objects at risk are the valued goods these individuals interpret to be endangered (e.g., wealth, prosperity, health). According to Boholm and Corvellec (2011), the relationships between risk objects and objects at risk are not simply “out there in the world”, but are socially constructed by an observer (involving, for example, imaginations, narratives, models, concerns, etc.), involve causal reasoning that links risk objects and objects at risk in a cause-effect relationship, and are tied to decisions under uncertainty (Boholm and Corvellec, 2011, pp. 180–181). In this latter sense, they are related to Luhmann’s (1993) account of risk, according to which risks are inextricably tied to decisions with uncertain outcomes and are thus observer dependent. For decision makers, a potential harm appears as a risk, whereas for those who are affected by the decision, this harm appears as a danger.

Although the relational theory of risk places great importance on the fact that both, risk objects and objects at risk, are socially constructed and culturally framed, it ultimately takes a cognitive view on the relationality of risk. The relations that this approach discusses are basically relations between cognitive objects, i.e., between representations of what is subjectively interpreted as hazardous and of what is perceived to be endangered. Arguably, objects at risk, risk objects and their relations are not idiosyncratic representations. Instead, they are social representations, they are socially constituted and shared. People tend to share what they hold dear and what they feel is endangered, for instance wealth, health, or social status. They also often concur in what they interpret to be hazards to these values, for instance climate change, economic downturn, or tobacco.

The present article builds on this understanding of risk and proposes to significantly extend the reach of the concept of relationality and thus of our understanding of the social nature of risk. Drawing on theories of relational sociology, we argue that risk is relational not only because of the observer-dependent links established between different cognitive objects, but also because risk in this understanding establishes *observer-independent social relations* between two or more actors. Risk thus becomes one specific way in which actors are linked to one another, a situation we call *risk entanglement*. We propose that this is the case when different actors are related to a common object, event, or a third actor in a way that involves risk and through these relations become risk entangled. Further, we propose that a specific case of risk entanglement occurs when different actors mutually construe each other as risk objects, a situation we call *risk reciprocity*. Typically, these constellations occur when there are substantial and institutionalized interactions, mutual dependencies and contingencies between actors, for example law-abiding citizens and criminal offenders, science and public health authorities, investors and companies, politics and finance. Reciprocity here does not imply equality or sameness in the degree of risk^1^, but rather a form of mutual interdependence. Importantly, this view is not limited to individual actors, but also encompasses collective actors (firms, organizations, corporations) and potentially larger social formations, such as fields or networks. Equally important, risk entanglement is in principle not limited to dyadic relations, but likewise stems from complex webs of social relations and more distant and indirect links between actors.

We suggest that the understanding of risk entanglement in contemporary societies is pivotal not only for more comprehensive theories of social fields and organizational behavior, but also for policy makers, organizations, and businesses who need to understand their entanglement in multiple risk relations. In the following, we first outline the existing “relational risk” approach in more detail and discuss how the concept of relationality can be meaningfully extended to also include social relations. We then propose a specific concept of relationality that draws on different accounts in relational sociology and field theory to give a precise account of our concept of risk entanglement. In a final step, we use data from 68 qualitative interviews and ethnographic fieldwork in the German financial sector and the political arena (henceforth the *finance-state nexus*) to lend empirical plausibility to our argument. We close with a summary and a brief discussion.

## The social relationality of risk

Relational understandings of risk have most prominently been articulated by Åsa Boholm (2003, 2015) and Hervé Corvellec (Boholm and Corvellec, 2011) in their relational theory of risk (RTR). RTR is rooted in classical cultural and interpretative conceptions of risk, as developed by Douglas and Wildavsky (1982). Both accounts criticize purely individualistic perspectives on risk, according to which risk ultimately is a matter of—idiosyncratic—subjective perceptions and interpretations. Instead, they argue that these perceptions and interpretations need to be understood with regard to broader social and cultural context, into which they are embedded.

The cultural theory of risk (Douglas, 1982) draws on a “grid/group” typology of institutional forms that can be used to describe the institutional make-up of different societies. The group dimension refers to the degree to which individuals are incorporated into social institutions and how these affect individual choice. The grid dimension represents how people’s lives in general are constrained by “externally imposed prescriptions” (Thompson et al., 1990, p. 5). Based on this typology, Douglas and Wildavsky (1982) developed an account of risk, and in particular of risk perception, according to which social institutions are the main forces drawing attention to some and diverting attention from other risks. How individuals perceive risk, and whether they perceive a situation as involving risk in the first place, therefore becomes much more a question of the institutions and cultural practices of a society rather than of individual perceptions of and preferences for risk. The cultural theory of risk has widely been interpreted as stating that the cultural, social, and institutional domains into which actors are embedded systematically affect how they perceive risk, thus inducing some form of cultural “risk bias” (see Johnson and Swedlow, 2021, for an overview).

A broad line of research has aimed at testing the propositions cultural risk theory and investigated associations between different institutional domains (according to the grid/group typology) and individuals’ risk perceptions (Boholm, 1996), finding only weak evidence for the hypothesized associations (e.g., Sjöberg, 1997; Slovic and Peters, 1998; Marris et al., 1998). This has led critics of cultural risk theory to suggest that typologies such as the grid/group distinction are probably too coarse to reliably explain risk perception.

Given the conceptual criticism and weak evidence for the hypothesized risk biases, a more recent strand in cultural theorizing of risk has suggested to not look at large scale institutional fields, but to instead focus on how culture and discourse create and define “risk objects”, i.e., things in the world that are widely—though not universally—perceived to be harmful (Hilgartner, 1992). What some people perceive as risky, and others might not, is thus not an inherent quality of these risk objects, but instead an outcome of how these objects are framed and constructed, and sometimes created, through cultural, in particular linguistic, practices. Climate change is an apt example: It often cannot be directly sensed or apprehended, but scientific evidence and political discourse render it a highly salient risk (e.g., Talwar, 2021). Most people acknowledge this framing, whereas others remain skeptical, which is why any process of constructing risk objects remains socially differentiated and disputed, even regarding the most obvious hazards, as COVID-19 has shown (Brown, 2020).

In the literature, this idea is frequently associated with the work of Hilgartner (1992), who elaborates on the construction of risk objects and their (causal) association with harm in sociotechnical contexts. This idea has been taken-up by Boholm and Corvellec in developing a relational theory of risk (Boholm, 2003, 2015; Boholm and Corvellec, 2011). Boholm and Corvellec (2011) follow Hilgartner in proposing that the cultural construction of risk objects is essential to understand the cultural dimension of risk. However, they extend this line of reasoning in proposing that it is not only risk objects that are imperative to understand risk perception, but at the same time “objects at risk” and the relations between them. Objects at risk are those things in the world that are imbued with value, that one holds dear, that are important to someone and that are threatened or endangered by a risk object. In the same way as risk objects are not simply objects deemed harmful by an individual, but are socially constructed and widely agreed upon to be harmful, many “objects at risk” are widely shared amongst groups and communities, some even being universal across the human species.

To complete this picture, Boholm and Corvellec (2011) emphasize that the relationship between risk objects and objects at risk is in itself an important part of the theory and cannot be taken for granted. Very generally, these relationships are assumed to be socially constructed by various means, they are “a semantic association between objects” (Boholm and Corvellec, 2011, pp. 180–181). These associations are built and constructed by observers through inferences, narratives, and models, they can be imagined, hypothesized or empirically verified. Three aspects of these observer-dependent relationships are noteworthy: First, they involve *narratives*, in particular narratives of probabilities. These narratives pertain, for example, to the risk object manifesting itself and coming into existence (e.g., a hurricane), the frequency or occurrence of an interaction between the two objects (e.g., brief or enduring exposure to a pathogen), or the magnitude of harm that is actually done (e.g., the cancerogenic nature of smoking). Boholm and Corvellec (2011, pp. 180–181) stress that these relationships are like narratives of what might hypothetically happen rather than narratives of what has actually happened. Second, relationships must involve *causal reasoning* in the sense of why and how a risk object can affect an object at risk. Causal reasoning can of course draw on different domains of knowledge, such as folklore or religion, but scientific evidence certainly assumes a privileged position here (Boholm and Corvellec, 2011, pp. 180–181). Third, relationships involve accounts of *agency*. A relationship typically involves portrayals of actions, practices, or decisions being implemented—or not implemented—that essentially contribute to (the probability of) the risk object being able to affect the object at risk.

Taken together, then, risk perception is strongly observer dependent and results “from situated cognition that establishes a relationship of risk linking two objects, a risk object and an object at risk, in a causal and contingent way so that the risk object is considered, in some way and under certain circumstances, to threaten the valued object at risk” (Boholm and Corvellec, 2011, p. 176).

## Extending the scope of the relational theory of risk

Although genuinely social and cultural in scope, the human being is the main site of those cognitions that specify the value-harm link involved in risk perception. The theory capitalizes on “situated cognizers” and individuated cognitive objects and structures that represent the different objects and the relationships between them. Arguably, as Boholm and Corvellec (2011) make unambiguously clear, there is much room for social and cultural processes in this picture: These cognitive representations are (a) typically not arbitrary and idiosyncratic, but they are—as any symbolic knowledge—socially constructed. They are (b) also assumed to be socially shared amongst segments of a population, for instance groups, organizations, or communities. Finally, (c) they are part of the symbolic order of a society, they are part of culture and discourse, that is they are articulated, negotiated, and formed through discourse and language in different domains, such as politics, religion, the economy, science, or the arts (e.g., Bouchet et al., 2022).

We propose that although this perspective is highly conducive to better understand the social and cultural dimensions of risk perception, it is somewhat limited given that its assumptions of relationality mainly focus on relations between (cognitive representations of) risk objects and objects at risk, putting less emphasis on the genuinely *social*, observer-independent relations that may ensue from these cognitive relations. In our view, RTR holds the potential, in an extended form, to make significant contributions to understanding not only “mental” and discursive relationships of risk, but also those risk-focused social relations that make-up much of contemporary, highly differentiated and networked societies. Specifically, we propose that principles of RTR can further our understanding not only of observer-dependent risk constructions and risk-related cognitions, but also of the manifold (observer-independent) risk relations (e.g., between actors, organizations, systems, or social fields) and the *risk practices* they entail, i.e., the distinct and often habitualized ways of acting upon, managing, and mitigating risk.

The notion of risk practices is particularly helpful to reconcile RTR’s emphasis on cognitive objects and structures and our proposed focus on social relations and situated risk behavior. In-line with the cultural theory of risk and Bourdieu’s (1977) original understanding, the concept of practice accounts for the view that (a) risk is contingent on internalized culture (as structured perceptions and cognitions), (b) that it is intricately linked to social fields and the relations that constitute these fields, and (c) that it includes an enactive dimension of oftentimes “habitual” risk behaviors.

To illustrate our proposed extension of RTR, we build on an example by Boholm and Corvellec (2011, p. 184), who describe the case of a railway tunnel through a ridge in Sweden. Because of complex geology and high groundwater pressure, groundwater at some point leaked through the tunnel walls. This leakage, firstly, endangered the tunnel’s overall integrity and, secondly, led to declining levels of groundwater in the area, making the above soil less fertile and less suited for agriculture. In this situation, the tunnel is an object at risk for the tunnel engineers, which is threatened by groundwater leakage as the risk object. For the local residents and farmers, however, the tunnel is the risk object (because it causes declining levels of groundwater) and their harvest becomes the object at risk (see also Boholm, 2008).

This triadic situation is not only characterized by relations between the cognitive representations (of risk objects and objects at risk) of the different actors involved, which RTR emphasizes, but it establishes new or alters existing social relations between actors: We emphasize that these observer-dependent constructions of risk translate into actual behaviors and practices and thus into objective, i.e., observer-independent social relations. It is only through their specific constructions of risk objects and objects at risk that railroad engineers and local farmers become socially related in the first place. In the example of the tunnel, these relations are established between human and non-human actors (e.g., between engineers and groundwater, or between local farmers and the tunnel), but also between human actors, i.e., between engineers and local farmers, all of whom become risk entangled.

### Risk entanglement

We therefore define *risk entanglement* as a situation in which a number of actors is, or becomes, related to each other through their constructions of risk objects and objects at risk. We use the term entanglement mostly in a “theory neutral” way, not explicitly drawing on, for example, its uses in quantum physics, history, or postcolonial studies. There is some overlap, however, to its uses in the latter two fields, where the term denotes being (often inadvertently) enmeshed or ensnared in complex sets of relations across human and non-human entities, which are “bound together” on both, material and symbolic dimensions (e.g., Nuttall, 2009; Giraud, 2019).

Along these lines, we use the term entanglement to signify the interrelatedness or interwovenness, at times the mutual dependency, of (a) the risk constructions of two or more actors and (b) how they become manifest in social relations and risk practices. Risk entanglement can take different forms. In Boholm’s and Corvellec’s (2011, p. 184) original example, two (groups of) human actors become related through a common object or non-human actor (the tunnel), which is their object at risk and their risk object respectively. Another example might be COVID-19, which can be considered a common risk object to politics and businesses. Although, and differently from Boholm’s and Corvellec’s example, both construe Covid-19 as a risk object, they retain distinct objects at risk, i.e., company revenue in the case of businesses and social order in case of politics. This is a case of risk entanglement because politics and businesses mutually affect one another when making decisions to avoid potential harm, which in turn might pose a danger to the respective other party (e.g., lockdowns, layoffs, ceasing operations).

In these examples, risk entanglement involves human actors and non-human entities, but we can intuitively think of other constellations that only involve human actors, both individual or collective. Risk entanglement therefore creates a situation in which two or more actors are not only related in terms of their perceptions and cognitive assessments of risk, but also in terms of how they act upon perceived risk, i.e., in their risk practices.

### Risk reciprocity

A specific case of risk entanglement in which risk practices become particularly relevant is a constellation we term *risk reciprocity*. This is a situation in which two or more actors mutually construe one another as risk objects. Initially, in Boholm’s and Corvellec’s (2011) example, the tunnel and the groundwater leakage are the risk objects. However, when engineers act upon the tunnel or the surrounding geology, this might produce the risk of groundwater levels declining further, which is why engineering work can become the risk object for local farmers. Conversely, local farmers might mobilize political protest and demand further reinforcement of the tunnel, which is costly for the railroad company, and makes local farmers the risk object of the company. In the COVID-19 example, politics not only frames the virus in a specific way, but also issues policies that impose restrictions upon businesses. Certain businesses might even be perceived as active drivers of the pandemic (e.g., cultural industries, sports events, tourism) and therefore be strictly regulated or even shut down. Politics thus becomes an even more obvious and severe risk object to businesses. In turn, businesses react in their specific ways to the virus and comply more or less stringently with political measures to contain the pandemic, some trying to evade regulations to maximize profit or to simply survive the pandemic. This way, also businesses become a risk object to politicians, because they can potentially undermine their efforts at mitigating the crisis.

In principle, this mirrors constellations of what Parsons (1951) called the “double contingency” of social interaction, which occurs when expectations concerning others’ actions “operate on both sides of the relation between a given actor and the object of his orientation, which distinguishes social interaction from orientation to nonsocial objects” (Parsons, 1951, p. 15). Although developed as part of his general theory of action and in principle unrelated to risk, Parsons’ perspective indeed does involve considerations of hazard, namely that social actors might infinitely dwell on expectations of others’ expectations of one’s own expectations (and so forth), risking the breakdown of cooperation (see also Vanderstraeten, 2002). In this sense, politics’ and businesses’ mutual orientation towards COVID-19 as a “nonsocial” object already establishes a form of risk entanglement between the two groups of actors, without involving a risk reciprocity relationship. Further down the road, however, they become risk entangled in a situation of double contingency and risk reciprocity, where the actions of one party become the risk object of the other party. Importantly, risk reciprocity (like double contingency) requires a relation between two (individual or collective) actors.

### Risk relationships and networks

For risk entanglement, not only the risk objects and the objects at risk are relevant in the manifold ways outlined by RTR, but also the specific risk relationships are. Actors engage and interact with each other not only based on their individual intentions, interests, and preferences, but also in terms of their relational positioning towards each other (e.g., in terms of roles, power relations, and status), their positions within network structures, and the cultural meanings attached to these dimensions (e.g., Fuhse, 2022). Risk in this perspective becomes an organizing force of relationality, i.e., an essential element in understanding how actors are related and linked to one another in society.

Empirically, dyadic risk entanglement will rather be the exception than the rule. How certain businesses perceive and act-upon the Coronavirus is not only a risk object for politics, but also for public health authorities. Likewise, strongly regulated businesses in the cultural industries of a city will be an object at risk for city officials because they contribute to the city’s reputation and tourism. Some actors or entities in the world can thus become the risk objects for a range of other actors for whom different objects at risk are at stake. This constitutes networked forms of risk entanglement.

In terms of general social theory, the novelty of this proposed extension of RTR therefore rests on an understanding of relations as not only pertaining to symbols or cognitive objects, but to individual and collective actors and larger social formations, such as Strategic Action Fields (Fligstein and McAdam, 2012). From the perspective of relational sociology, social order is not considered an established structure, but rather as constantly “in the making”, something that needs to be investigated in terms of processes, interactions, and social relations (Dépelteau, 2018). Importantly, actors in this perspective do not exist prior to or independently of their relationships to others, but are mutually constituted through these relations (Emirbayer, 1997, p. 296). Risk relations, as a particular kind of relation, are therefore constitutive elements in the “becoming” of actors, they not merely interlink actors as static entities, but they constantly *make* and *re-make* actors.

## An empirical approach to risk entanglement

Although our proposal for an elaboration of RTR might seem plausible in theoretical terms, this section aims at providing empirical insights into concrete and observable constellations of risk entanglement. Even though the research we describe here was informed by existing theories of relational risk, it significantly contributed to advance RTR theorizing and to the development of our proposed concept of risk entanglement. The original research aimed at investigating risk practices within *and* risk relations between actors in two Strategic Action Fields (Fligstein and McAdam, 2012), the state and the financial sector. Both have classically been described as key domains in which risk is produced and managed (Luhmann, 1993, pp. 145–86). More recent developments, in particular the global financial crisis 2007–2009 and the European debt crisis beginning in 2010, have pointed out the importance of understanding the risk relations between actors in these fields and how these developments have challenged established risk between them.

Investigating risk practices and relations in the finance-state nexus, we capitalized on how actors in both fields mutually observe, make sense of, and evaluate each other’s risk-related expectations, actions, and routines. A key assumption was that these risk practices are constantly produced and re-produced in the manifold interactions and relations between the two fields. These aims as well as the general approach of a relational theory of risk called for a primarily qualitative-interpretive methodological approach to reconstruct how actors create the relationship between risk objects and objects at risk and thereby also inform the respective institutionalized and collective dimensions of risk. Therefore, we conducted a study of the German finance-state nexus between 2017 and 2020 using narrative, problem-centered interviews (Witzel and Reiter, 2012), as well as ethnographic observations (Hammersley and Atkinson, 2007) to investigate actors’ interdependencies, mutual interpretations and attributions, and interlaced or even joint practices. Specifically, we opted for the comparative approach emphasized in Grounded Theory Methodology (Corbin and Strauss, 2015), which required using the same principles of data acquisition and analysis for actors in the state and finance field.

### Sample

We conducted a total of 68 interviews in the German financial and state fields to reconstruct respondents’ occupational practices, paying particular attention to experiences and reflections of crises and concepts of risk and risk management. This allowed us to uncover genuinely relational aspects of risk, for instance when financial actors articulate how perceptions of and interactions with state-bound actors affect their constructions and perceptions of risk—and vice versa.

Twenty-five interviews were conducted in the German financial sector, with respondents working in private, cooperative, and public savings banks as well as in insurance companies. Respondents worked mainly in risk management, bond trading, derivative investments, and quantitative engineering and research. Our sampling strategy aimed at gathering a diverse range of actors representing potentially different risk practices within the financial sector, such as quantitative and qualitative risk management.

A further 26 interviews were conducted with actors in politics and government, covering both federal and state jurisdictions. This included political representatives (members of the federal and state parliaments) working in the fields of finance, household, and the economy who typically deal with issues of financial regulation, debt management, and fiscal policy. Respondents were mostly members of the budget and the financial committees of federal and state parliaments. In order to examine finance-related risk practices also in terms of their genuinely political, and not necessarily financial elements, we occasionally examined and contrasted these with risk practices in other jurisdictions, for example environmental and cultural affairs.

We also conducted 17 interviews in the state executive branch to more precisely acquire narratives of individuals who are directly concerned with financial and regulatory entanglements of the state and finance. This included, for example, regulators, financial supervisors, and researchers at the German Financial Supervisory Authority, the German central bank Deutsche Bundesbank, and treasurers in federal and state executive organizations responsible for issuing government bonds. Finally, we observed ethnographically, over the course of a year^2^, public and closed meetings of the Finance Committee of the German Bundestag to assess the interactive and situated dimension of risk relations. Taken together, this sample allowed us to look at risk relations and practices as well as constellations of risk entanglement within and across the fields of state and finance. The transcribed interviews and ethnographic field notes were analyzed in an integrated manner using Maxqda software and the coding principles of Grounded Theory (Corbin and Strauss, 2015).

## Risk entanglement in the finance-state nexus

Taking the finance-state nexus as an arena for an inquiry of risk entanglement needs to acknowledge that the state and finance are two historically highly intertwined fields that have recently been dealing with substantial challenges related to risk and risk management (specifically due to the financial and the European debt crises). In analyzing our data, we focus on a “regulation dilemma” that is characteristic for the finance-state nexus to explicate how actors become risk entangled in different ways. In particular, we elaborate on how actors in both fields mutually construe each other as risk objects, thereby illustrating dyadic risk reciprocity between two fields. We also focus on the implications these construals bear for the respective objects at risk and the relations between these fields and actors.

This dilemma shows that the state is confronted with the challenge to curtail certain financial activities, such as excessive trading or use of overly complex financial instruments, while at the same time facing the need to safeguard a healthy financial system to re-finance itself (e.g., through government bonds), to provide liquidity to the real economy, and to secure pension funds. Regarding financial actors, this dilemma poses a constant threat to their goal of generating liquidity and profit. To this end, financial actors constantly evaluate the reliability and solvency of states. The regulation dilemma thus is a prototypical situation of relational risk, that is, of mutual risk attribution and assessment between the state and the financial sector.

**Fig. 1:**
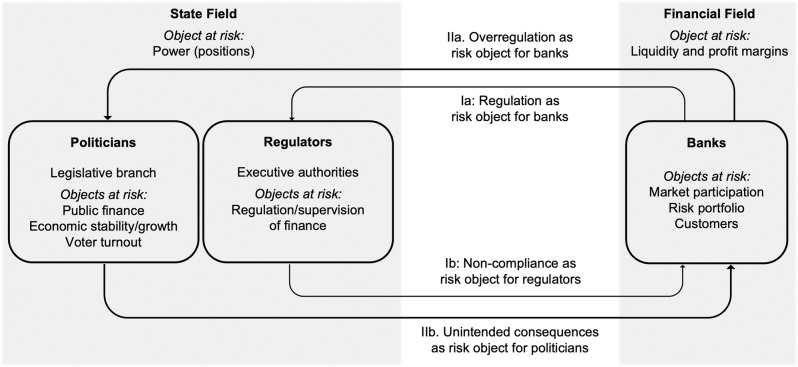
Risk entanglement in the finance-state nexus. Arrows point from actors who construct towards those who are constructed as risk objects.

Referring to the theory of Strategic Action Fields (Fligstein and McAdam, 2012), we empirically assess these risk relations in a model of risk entanglement in the finance-state nexus (see Fig. 1) to analyze how actors in these fields mutually construe each another as risk objects with respect to their field-specific objects at risk.

Looking at state actors, our data show a range of different risk objects that endanger several objects at risk in the state field. Even with respect to financial risks, state actors engender a broad range of risk practices. For example, assessing financial liquidity issues requires entirely different risk constructions and evaluations than dealing with dangers to establish a “green” financial industry. However, our analysis of the broad variety of risk constructions reveals a main category of objects at risk in the state field: maintaining political power, both in terms of state, government and administrative power and with regard to the power of parties and political fractions. Two types of actors are particularly relevant here, politicians and regulators. Politicians are essential to legislation. They pass legal bills and regulations at national and supranational levels. Their specific objects at risk are their own positions and mandates as well as voter support. In many cases, and particularly in view of the recent crises, this means curtailing financial markets while at the same time securing the state’s budget, keeping-up liquidity for the real economy, and securing market-based pensions. Regulators typically are employees in government executive branches and organizations and their objects at risk are the successful enforcement and monitoring of banking and finance directives.

In the field of finance, we also find several risk practices that can be differentiated, for example, along financial domains (e.g., bonds, stocks, or derivatives) and typical risk constructions related to these domains, such as quantitative or qualitative risk management. However, this diversity in risk constructions can be subsumed under a more general category of endangered financial objects at risk: the flow of payments that generates profit, culminating in particular variants like managing market participation, risk portfolios, and customer mandates.

### Regulation as a risk object for banks—non-compliance as a risk object for regulators

Regulators in the state field ascribe banks the key competence for taking and managing financial risk. This is why the general approach of regulators and authorities is not to manage financial actors’ risks, but to oversee whether risk management is “reasonable”, as described by one supervisor, who also states that it is still “the task of a bank to take risks”. Besides the principle of “double proportionality”, which implies that larger firms holding greater risks are monitored more stringently than smaller and less risky ones, a “dialogical approach” characterizes contemporary financial supervision in Germany as a paradigmatic risk relationship between regulators and actors in finance. This principle implies a shifting between mandatory rule compliance and a continuous dialog with financial actors who, ideally, should “work peacefully and let us administer them as comfortably as possible”, as expressed by another supervisor. The dialogical approach thus creates a certain leeway for regulators and banks, but also mandates that suspicious cases are assessed meticulously. This approach goes hand in hand with the threat of possible sanctions. The risk practices of the supervisors we interviewed rely on the general assumption that financial actors are well aware of this principle. A supervisor describes this risk relationship metaphorically when suggesting that it is not the “forester” (i.e., the supervisor) who keeps the forest tidy, but rather banks’ fear of the forester:

[…] it’s not the forester who keeps the forest in order, but the fear of the forester. [laughs] So that makes it clear what I mean. Just that there is […] banking supervision. And that it has a certain set of instruments already leads to a certain disciplining. […] So, if I see that the forester is walking back there, […] I take my garbage home. […] I don’t wait until the forester comes and catches me.

This constellation represents one direction of risk reciprocity: Regulators become risk objects for bankers because they have the power to curtail and therefore endanger the margin-related leeway through direct interventions into banks’ operations. There is broad consensus among the financial actors we interviewed that the current regulatory framework did indeed stabilize the financial sector. Financial actors also predominantly recognize the need for stronger regulation, including regulatory directives in their own operations.

Thus, the principle of dialogical and proportional regulation leads to uncertainties between regulatory demands and the degrees of freedom to implement these demands, which qualifies financial oversight and regulation as significant financial risk objects. The following quote of a risk manager in a savings bank illustrates this uncertainty:

The requirements of the supervisory authority on the subjects of ‘deal with your risks, that’s your job,’ of the proportionality approach, the ‘MaRisk’ [national manual on risk management] […]. It was meant to be great, but it was implemented disastrously, because as soon as a bank thought about it, the methodologists came and said, ‘You can’t do what you’re doing'. Then come the associations, then come the politics, then come the technology and everything else. […] You have no chance / to apply what […] is made with sense and reason to suit you and you accordingly have a good feeling there and name the result as correct, for you as correct and effective in terms of control, in terms of supervisory law.

This quote suggests that acknowledging banks’ independence in risk management, as demanded by the supervisory authority, is doomed to fail since it necessitates proprietary tools and procedures, which are too frequently criticized or rejected straight away by the supervisory authority. The demand for “reasonable” risk management, as stated in the above quote, is in principle acknowledged by the banker, but in his view hardly ever leads to a satisfactory outcome because authorities tend to intervene, which creates a control problem. The risk manager notes that, ideally, his department would therefore have to be “duplicated” and “all work would have to be done twice. Once in the way the authority wants it done, and once in a way you can control”. Thus, the regulator as a risk object is perceived as a concrete threat challenging financial actors’ own objects at risk. In addition to dealing with challenges of credit or interest rate risks, financial actors also need to deal with regulation as a further type of risk, namely an audit risk stemming from the need to decide whether to comply with or try to evade regulation.

The decision the banker needs to accomplish represents the second direction of this risk reciprocity relation: The banker’s evaluation becomes the regulator’s risk object. For the latter, non-compliance is in fact a threat to successful supervision and regulation (and thus ultimately to state executive power) as an object at risk. A typical risk faced by regulators is to fail to anticipate rule evasion. A supervisor describes this risk:

And we clearly take the risk of missing something, that’s just the way it is. There’s so much complexity, whether it’s insurance or banking, somebody always slips through the cracks.

He acknowledges that there will always be financial actors who get away with bypassing regulatory requirements and that it is in principle impossible to supervise all financial actors with regard to compliance.

Taken together, these portrayals of regulatory risk illustrate how regulators and bankers construct each other as risk objects and thus as dangers that challenge their respective objects at risk. Therefore, as a consequence of ex-post crisis management, bankers and regulators are not simply embedded in a permanent flux of interactions, but are substantially related in constellations of risk entanglement. Importantly, these entanglements are not only cognitive or discursive constructions (of one actor being the risk object of the other actor), but they imply and constitute specific forms of how regulators and financial actors relate to and interact with each other.

### Unintended consequences as a risk object for politicians—over-regulation as a risk object for banks

Looking at risk reciprocity between bankers and politicians, the relationship between the need for regulation on the one hand and the resulting organizational and procedural uncertainties on the other hand becomes even more evident. This culminates in a general regulatory dilemma as a central source of uncertainty among the politicians we interviewed: On the one hand, they are supposed to contain systemic risk in the financial sector to prevent an economic meltdown. On the other hand, they are supposed to maintain a well-functioning financial sector.

The challenges of political risk management therefore pertain to pursuing the aim of risk minimization, which, however, tends to run counter to the ascribed risk function of the financial sector as a dedicated “high-risk” arena. Many of the politicians we interviewed highlight a fundamental problem: intervention into complex financial practices, which is necessary to achieve risk minimization, can lead to consequential risks as unintended consequences—that is, they create risk through risk management (Holzer and Millo, 2006; Luhmann, 1993; Power, 2009; Rothstein et al., 2006). Financial actors therefore become risk objects for politicians in at least two interrelated ways: On the one hand, this concerns the state’s dependence on financial liquidity and hedging against temporal uncertainties. On the other hand, this concerns an extension of the regulatory risk of non-compliance among financial actors. From this perspective, however, not the above-mentioned regulatory risk is paramount, but rather “niche-seeking” among financial actors as a result of the cutting off from financial leeway or the containment of entire markets (e.g., non-transparent over-the-counter transactions, real estate, “creative finance”).

The following quote by a member of the German Federal Parliament clarifies this as an expectation towards financial actors. Through the metaphor of the “shotgun in the fog” it also illustrates the problem of regulatory proportionality:

[…] the way it is with regulation is that you just shoot the shotgun into the fog first and hope you hit the right guy. Whether you hit the right one always turns out afterwards. Because these complex systems naturally develop circumvention strategies. In other words, you have to consider in what you do what possible circumvention strategy there might be, and doesn’t it create even more risks than what we have now? Only, of course, we don’t manage to develop all possible circumvention strategies now, in our imaginary world, because that’s simply not possible.

Political regulation is thus confronted with the problem that it basically has to address the entirety of the financial sector, even if in principle only a few actors are inclined to not comply. It is then up to ex-post analyses to determine whether “the right one” is affected by regulations and financial risks have in fact been successfully mitigated or whether new risks have emerged. This prompts the question of over-regulation: The regulatory dilemma for politicians thus consists in having to develop, implement and enforce regulatory directives while at the same time avoiding over-regulation as a stalling of (financial) economic activity.

The danger of over-regulation is thus also the essential criterion for identifying politicians as risk objects for financial actors. Our analyses show that the restrictions of financial leeway as well as the practical challenges and monetary expenditures linked to them threaten the provision of liquidity and profit margins as key financial objects at risk. Bankers’ evaluations of present regulatory practices such as “bypassing the market” or “well-intentioned, but perhaps simply going too far” thus also indicate threats to market participation, risk portfolio management, and customer relations as specific objects at risk for banks.

In addition to the expectations of politicians towards financial actors, which include the acknowledgment of potential over-regulation and the possible development of circumvention strategies, financial actors also hold expectations regarding the state: namely, the necessary curtailing of regulation (or even deregulation) rooted in the state’s dependence on a functioning financial economy. This understanding, as well as the acknowledgment of the need for regulation, is expressed in the following quote from a banker:

And that is why it is indeed the case that some states in particular are very dependent on the banks holding this on their balance sheet, […] and at the moment it is the case that the banks, in the current phase they are in, need the states less. Now, one can argue: Yes, if the state had not helped in the financial crisis, there would not be many. That is correct. In fact, the state was important in stabilizing the system, and the system as a whole, or at least many of the players, would no longer be on the market in this form if the state had not intervened. Therefore, you have to distinguish between periods and points. Nowadays, it’s rather, I would say, that for the refinancing of the sovereigns, the banks are very sustainably important, frankly.

It is only through these interrelated, longer-term expectations that risk entanglement between financial and state actors can be understood comprehensively. These interrelated, longer-term expectations constitute a type of risk entanglement that adds to the types discussed in the previous section. Over-regulation and unintended consequences are two examples of risk objects that contribute to the shaping of more enduring social relations between the state and financial actors. These risk relations are characterized by anticipations and assessments of possible future behaviors of both actors and the consequences these behaviors might imply—for the actors but also for society at large.

## Conclusion

Risk profoundly pervades human social life. This has become most evident during the COVID-19 crisis, but is equally apparent looking at past events, such as the financial crisis 2007–2009 or the Fukushima nuclear accident in 2011. Social science approaches to risk for a long time have argued that risk should not only be understood as an individual, psychological phenomenon, but needs to be comprehended in terms of its social construction and its social consequences, for example regarding cultural differences in risk perception or the manifold political and economic attempts at risk management and mitigation. The relational theory of risk is one such approach, proposing that risk can be understood as observer-dependent relations between risk objects and objects at risk. The present contribution aimed at further developing this relational account of risk. Whereas the relational theory of risk mainly focuses on how individuals establish interrelated representations of risk objects and objects at risk and how these (semantic) interrelations are constructed in discourse, we argue that risk also establishes genuinely *social* (i.e., observer-independent) relations between actors or entire social fields that are different from the sorts of cognitive relations emphasized by the relational theory of risk.

This idea is reflected in our concept of *risk entanglement*, defined as a situation in which a number of actors is, or becomes, related to one another through their risk assessments. We have discussed different forms of risk entanglement, for instance constellations in which one and the same object or event is a risk object for one party and at the same time an object at risk for another party (Boholm and Corvellec, 2011). Furthermore, risk entanglement also encompasses constellations in which an actor or event is a common risk object for two different parties. In these cases, while both parties perceive such a risk object as threats to their (potentially) distinct objects at risk, it is very likely that the common risk object socially relates these parties to one another, thus establishing a new or altering an existing (risk) relationship. Such constellations already create situations of double contingency. However, this becomes even more evident in situations in which two actors mutually construe one another as risk objects, which we label *risk reciprocity*.

We have argued for the plausibility and empirical applicability of our approach using data from a study on risk practices in the German finance-state nexus. Our analysis indicates that the manifold relations between the fields of finance and state can be meaningfully described and understood in terms of their risk relations. With respect to market-based public debt management and the regulation of finance, our analysis of the “regulation dilemma” shows how bankers and regulators as well as bankers and politicians become risk objects to one another, thus mutually threatening their respective objects at risk, i.e., maintaining power (politicians, regulators) and profitable payments (bankers). Therefore, financial and state actors are not only embedded in several financial, fiscal, regulatory, or political interdependencies, but are substantially related in constellations of risk entanglement (see Lange and von Scheve, 2022).

Our theoretical and empirically informed proposal can be extended in at least five ways, outlining fruitful avenues for future research. First, it has become evident that risk entanglement can occur at different levels of collectivity. It may involve individuals and small groups who are capable of coordinated social action. This would also include other forms of social organization, such as communities, corporations and organizations. Potentially, risk entanglement can be meaningfully described not only regarding more narrowly defined Strategic Action Fields, but also regarding social fields more generally. Risk entanglement therefore becomes a function of how social collectives are organized, of their communicative infrastructures, the degree to which they share objects at risk, the consensus in understanding a specific risk relationship, their potential for collective action, and their specific ways of understanding, addressing and managing risk.

Second, collectivity almost always implies some degree of institutionalization that governs risk relationships and that may bring about risk objects and objects at risk that are germane to specific institutional domains. Institutions typically involve systems of formal and informal rules, tacit knowledge and habits that govern social interactions. The degree of institutionalization grows with increasing levels of collectivity. Collective actors such as organizations and corporations are typically rooted in specific institutional domains, such as politics, the economy, or science, all of which are characterized by rules governing interactions. Relations within and across institutional fields may be genuinely related to risk and will most likely differ in terms of the risk practices that evolve within a field.

Third, risk entanglement is likely to be networked in various ways. Actors are typically embedded into a multitude of risk relations at the same time, each potentially affecting one another’s risk assessments. From this perspective, risk entanglement can be described horizontally, for example in terms of reachability in a social network, and vertically as a hierarchically layered phenomenon, for example when investigating forms of risk entanglement within an organization (such as between departments) and between different organizations. Furthermore, risk reciprocity relations can create network constellations that resemble Granovetter’s (1973) “forbidden triad”: when two actors entertain risk reciprocity relations with a common third object or actor, it is highly unlikely that the two are not risk related in some way.

Fourth, risk entanglement is likely to be *interlaced* to varying degrees, differing in terms of immediacy and situatedness, directness and indirectness, involving holes, bridges, synchronous as well as asynchronous interaction. Risk relationships may occur fleetingly, in spontaneous and unpredictable ways, such as in brief face-to-face encounters, or they may be part of enduring and institutionalized relationships that carry strong and discursively framed meanings and practices related to risk. Actors, groups, or social fields can become one another’s risk object even without ever interacting directly with each other, but rather because they know that another actor’s actions may feed into a complex network as risk objects, thus affecting, directly or indirectly, a multitude of other actors with various objects at risk—and vice versa.

Fifth, risk entanglements are likely to evolve over time. They are not static because they are closely linked to social relations, which change over time for various reasons. Some of these reasons can be found in the previous four avenues for future research: the structure and composition of collectivities vary across time, institutions undergo intentional and unintentional changes and revisions, social networks shift and evolve regarding their ties and nodes, and interlacing is in itself a time-varying factor.

Taken together, the proposed understanding of relational risk and risk entanglement should offer a range of conceptual tools to further our understanding of the complex risk relationships in contemporary societies. It emphasizes the ways in which individual and collective actors as well as social fields are mutually interdependent when assessing and managing threats to valued goods or when they pose threats to one another. From the perspective of relational sociology, these complex interdependencies do not merely “affect” the actors involved, but risk entanglement from this vantage point is constitutive for them.

## Data Availability

To safeguard agreed-upon participant anonymity, only excerpts of the data can be partially made available from the authors upon request.

## Footnotes

1 This is typically implied in uses of the term “reciprocal risk” in law (e.g., Fletcher, 1972).

2 We cannot disclose the exact year because of anonymity requirements.
